# Physical activity may a probably protective factor for postoperative delirium: the PNDABLE study

**DOI:** 10.3389/fnagi.2024.1353449

**Published:** 2024-04-03

**Authors:** Jian Kong, Xu Lin, Bin Wang, Shanling Xu, Yuanlong Wang, Shuhui Hua, Hongyan Gong, Rui Dong, Yanan Lin, Chuan Li, Yanlin Bi

**Affiliations:** ^1^Department of Anesthesiology, Qingdao Municipal Hospital, Qingdao, Shandong, China; ^2^School of Anesthesiology, Shandong Second Medical University, Weifang, China; ^3^The Second School of Clinical Medicine of Binzhou Medical University, Yantai, China

**Keywords:** physical activity, postoperative delirium, postoperative complications, Alzheimer-related biomarkers, cerebrospinal fluid

## Abstract

**Objective:**

This study aims to explore the relationship between physical activity (PA) and postoperative delirium (POD).

**Methods:**

We selected 400 patients from the Perioperative Neurocognitive Disorder and Biomarkers Lifestyle (PNDABLE) database, and the patients in the PNDABLE database were sampled and tested Alzheimer’s biomarkers. The diagnosis of POD was made using the Confusion Assessment Scale (CAM) and the severity was assessed using Memorial Delirium Assessment Scale (MDAS). Mini-Mental State Examination (MMSE) scale was used to detect the mental state of the patients. Enzyme-linked immunosorbent assay (ELISA) was used to detect the level of preoperative cerebrospinal fluid (CSF) biomarkers, such as amyloid β plaque 42 (Aβ42), total tau protein (T-tau), and phosphorylated tau protein (P-tau). Logistic regression, sensitivity analysis, and *post hoc* analysis were used to explore the relationship between risk and protective factors on POD. We used the mediating effect to explore whether PA mediates the occurrence of POD through CSF biomarkers.

**Results:**

The incidence of POD was 17.5%. According to our research, the consequence prompted that PA might be the protective factor for POD [odds ratio (OR): 0.336, 95% confidence interval (95 CI) 0.206–0.548, *P* < 0.001]. The result of logistic regression revealed that CSF biomarker Aβ42 (OR: 0.997, 95 CI 0.996–0.999, *P* < 0.001) might be a protective factor against POD, and the T-tau (OR: 1.006, 95 CI 1.003–1.009, *P* = 0.001) and P-tau (OR: 1.039, 95 CI 1.018–1.059, *P* < 0.001) might risk factors for POD. Sensitivity analysis confirmed the correlation between PA and CSF biomarkers in the patients with POD. Mediation effect analysis showed that PA may reduce the occurrence of POD partly through CSF biomarkers, such as Aβ42 (proportion: 11%, *P* < 0.05), T-tau (proportion: 13%, *P* < 0.05), and P-tau (proportion: 12%, *P* < 0.05).

**Conclusion:**

Physical activity is probably a protective factor for POD and may exert a mediating effect through CSF biomarkers.

## Introduction

Postoperative delirium (POD) is a serious complication with an acute change in cognition and attention, clinical symptoms may include altered consciousness and confusion (Rudolph and Marcantonio, 2011; Zenilman, 2017). The incidence of POD varies with the type of surgery, the urgency of the surgery, and the type and sensitivity of delirium assessment (Zenilman, 2017). Moreover, POD is a common postoperative complication in patients, and it is associated with adverse events such as functional decline, prolonged hospitalization, and risk of hospitalization (Dasgupta and Dumbrell, 2006; Sadeghirad et al., 2023). According to the poor cognitive and organic outcomes mentioned above, and convincing increased mortality that proposed in early research (Tune, 1991), we need to understand the inner workings of POD, and take active measures to reduce the incidence of POD.

Although the mechanism of POD is currently unknown, there is growing evidence that elements such as blood–brain barrier damage, CSF biomarkers, and neuroinflammation play an important role in the development of POD (Querfurth and LaFerla, 2010; Idland et al., 2017; Huang et al., 2018). Among the mechanisms mentioned above, CSF biomarkers have been extensively studied, such as amyloid β plaque 42 (Aβ42), total tau protein (T-tau), and phosphorylated tau protein (P-tau) (Körtvélyessy et al., 2015; Wang and Mandelkow, 2016). As an independent predictor, the level of Aβ42 may prognosticate the occurrence of POD and may be considered as a protective factor (Wu et al., 2023a). Meanwhile, T-tau and P-tau proteins might be risk factors for POD (Nizynski et al., 2017). Therefore, the above conclusions may be intertwined to conclude that a portion of CSF biomarkers are associated with POD.

Recent studies show that PA therapy was widely used in clinical applications, which is also applied in rehabilitation, such as improving the discovery of patients with neurological diseases (Tollár et al., 2020; Loftus et al., 2022). Epidemiological studies have shown that PA reduces the risk of cognitive dysfunction (Hötting and Röder, 2013; Lee et al., 2019). However, there is currently little research on whether PA and frequency of PA are associated with POD and the mechanisms involved. Considering the current PA status, actively exploring the relationship between PA and POD may be indispensable to reduce the occurrence of POD.

Referring to previous research, we have proposed a hypothesis that PA may reduce the incidence of POD, and we also make further validation of the hypothesis from the following questions. First, whether PA independently affects POD; second, if the former problem is confirmed, weather the frequency of PA has an impact on the incidence of POD; third, whether PA is related to CSF biomarkers; last, if patients who participate in PA occurs less with POD, whether it mediated through CSF biomarkers?

## Materials and methods

### PNDABLE database

Participants in this study were recruited from the Perioperative Neurocognitive Disorder and Biomarkers Lifestyle (PNDABLE) database, which is a large-scale ongoing cohort study, concentrating on the risk factors and biomarkers of perioperative neurocognitive disorder (PND) in the Han population of northern China, all participants were aged between 40 and 90 years old. This study has been approved by the Ethics Committee of Qingdao Municipal Hospital and registered with the Chinese Clinical Trial Registry (Clinical registration number of PNDABLE: ChiCTR2000033439).

All participants were informed and agreed to participate, and they were not restricted and could withdraw from the research at any time. CSF biomarkers and blood samples from all participants were used for future research.

### Participants

We chose to conduct this study in Qingdao Municipal Hospital, using the PNDABLE database from August 2020 to August 2021 as the source of information. Patients who met the following criteria were selected to participate in this study. Inclusion criteria include age 40–90 years old; underwent hip or knee arthroplasty; combined spinal and epidural anesthesia; ASA classification American Society of Anesthesiologists (ASA) I–II. Exclusion criteria were listed as follows: (1) preoperative Mini-Mental State Examination (MMSE) score <24 points; (2) drug or psychotropic substance abuse; (3) including long-term use of steroids and hormone drugs; (4) severe visual and hearing impairment; (5)preoperative coagulopathy; (6) severe systemic diseases (such as malignant tumors) may affect AD biomarker levels in CSF; (7) major mental illness; (8) resulting in language communication barriers; (9) patients who are bedridden for a long time and unable to perform PA due to disability; (10) genetic or family history; and (11) patients who were transferred to ICU postoperatively.

### Neuropsychological testing

During these evaluations, occurrences of POD were recorded using the Confusion Assessment Scale (CAM). Those who tested positive were categorized into the POD group, while participants with negative results were placed in the non-POD group (NPOD).

Additionally, the severity of POD was assessed using the Memorial Delirium Assessment Scale (MDAS) (Inouye et al., 1990; Schuurmans et al., 2003).

All of the above evaluations were performed by a group of physicians, including an anesthesiologist and a neurologist. The results of the patient’s preoperative evaluation were unknown, and the preoperative and postoperative evaluation of the patient was conducted by two different groups of doctors. CAM and MDAS are used in patients with a probability of reliability and universal applicability (Leung et al., 2008; Shi et al., 2014).

### Anesthesia and surgery

Participants underwent hip or knee arthroplasty surgery with combined spinal and epidural anesthesia. Prior to the operation, patients abstained from medication and strictly fasted from food and water for 6–8 h.

Upon admission to the operating room, routine monitoring including ECG, oxygen saturation, arterial blood pressure, and intravenous access was established. The puncture site was at the lumbar 3–4 space (L3–L4). Following successful puncture, 2 ml of cerebrospinal fluid (CSF) was collected from the subarachnoid space for biological detection indicators, and 2–2.5 ml of Ropivacaine (0.66%) was injected within the subsequent 30 s.

During the operation, the patient’s anesthesia level was controlled below the level of lumbar spine 8 (T8). We regularly checked the patient’s blood pressure, heart rate, oxygen saturation, etc. every 3 min and oxygen was administered to the patients via a mask at a rate of 5 L/min during the operation. After the operation, the patient will be sent to the anesthesia recovery unit (PACU) for observation for 30 min. The patients whose vital signs are normal will be returned to the ward.

### CSF core biomarkers measurements and collection

Two milliliters CSF was collected in a polypropylene centrifugal tube, then centrifuged at 2000 × *g* for 10 min at room temperature (Lundström et al., 2003; Bakr et al., 2017) as well as separated and stored in an enzyme-free EP (Eppendorf) tube (oxygen bottle, PCR02-C) at −80°C for further use in the following steps of this study.

Enzyme-linked immunosorbent assay (ELISA) was used to detect the level of Aβ42, T-tau, and P-tau, which were detected from 2 ml CSF, using Aβ42, P-tau, and T-tau assay kit under the manufacturer’s instructions (Thermo Scientific, Multiskan MK3). The concentrations of Aβ42/ T-tau and Aβ42/ P-tau were subsequently calculated. All samples were measured by the same laboratory personnel, and the group assignment blinded them.

### Classification of physical activity

We tracked the past PA status of the included patients by asking the patients just before the surgery, and divided the patients into two categories: physical activity (PA) or inactive. PA can include not only sports such as cycling, swimming, and tennis, but also farm work, physical work, and purposeful walking. Then the patients were classified into different groups according to the frequency of PA, and numbered the groups. The details are as follows: 0 = I participate in PA daily; 1 = I participate in PA multiple times a week; 2 = I participate in PA once a week; 3 = I occasionally participate in PA; 4 = I never participate in PA.

### Sample size estimation

The preliminary test in this study explored that eight covariates were included in the Logistic regression. According to the previous studies, the POD incidence was 17.6%, and the loss of follow-up rate was assumed to be 20%. Thus, according to the logistic regression events per variable (EPV) sample size calculation method (van Smeden et al., 2019), EPV set to 10, the required sample size was 568 cases (8 × 10 ÷ 0.176 ÷ 0.8 = 568).

### Statistical analysis

First, the K-S test was used to verify whether the continuous variables conform to the normal distribution, the data that fit the normal distribution are represented by the mean ± standard deviation (SD), and the data that do not conform to the normal distribution are represented by the median and interquartile ranges (M, Q25, Q75). Two independent samples *t*-test was used to test whether there were significant differences in CSF biomarkers and PA between the POD and NPOD groups.

Logistics regression was used to discuss if PA can affect POD independently and whether PA is a risk or protective factor on POD. Logistics regression analyses were also performed on the frequencies of PA further to explore the effect of different PA frequencies on POD.

Subsequently, to make the results more accurate, we further investigated the role of confounding factors in sensitivity analysis. Two steps of sensitivity analysis were conducted in this study, in the first step, we selected gender, years of education, and MMSE as the confounding factors, and in the second step, gender, hypertension, diabetes, coronary heart disease, smoking history, alcohol history, years of education, and MMSE were included in the sensitivity analysis.

Taking it a step further, we used a mediating effect model to examine whether PA influences the development of POD through CSF biomarkers. Only by following four key criteria satisfied simultaneously can the model be constituted: (1) association receded between POD and PA when the mediator (CSF biomarker) was included in the regression model; (2) a significant relationship between CSF biomarker and POD can be observed; (3) conspicuous correlation between PA and POD is noticed; and (4) connection of PA and CSF biomarker is remarkable.

*Post hoc* analysis was applied to exclude the patients with MMSE < 28, diabetes, and coronary heart disease to explore if the findings hold in the population who have better cognitive functions, without coronary heart disease, and have no diabetes.

SPSS 25.0 (IBM SPSS Inc., Chicago, IL, USA), R software version 4.3.1 (R Foundation for Statistical Computing, Vienna, Austria), and Stata MP16.0 (Solvu Soft Corporation, Inc., Chicago, IL, USA) were used for data analysis. The standard with significance was *P* < 0.05.

## Results

### Participant characteristics

**FIGURE 1:**
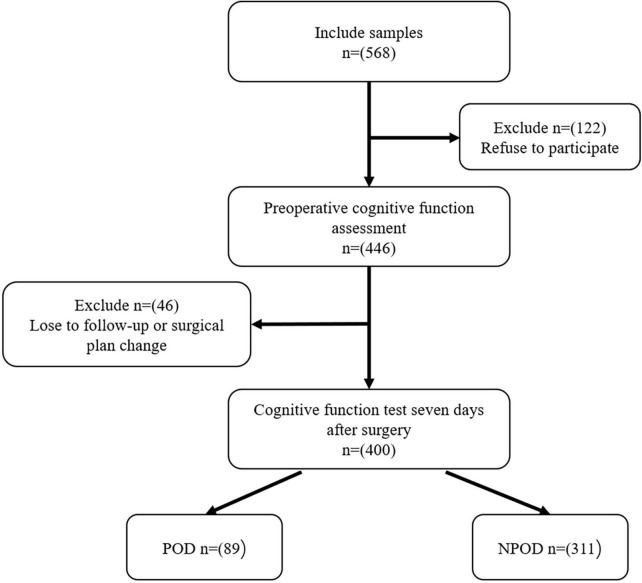
The flow diagram showed the selection of eligible patients and the enrollment process.

We included a total of 400 patients for final analysis after exclusion (Figure 1, flow diagram).

Of all the participants in this study, 89 (positive rate of POD: 22.3%) were diagnosed with POD within 1–7 days postoperatively, with a median age of 74 years. Participants were divided into two groups (POD and NPOD) based on whether they were diagnosed with POD. The results showed that there was significantly different in CSF biomarkers (Aβ42, T-tau, P-tau, Aβ42/T-tau, and Aβ42/P-tau) (*P* < 0.01), PA (*P* < 0.05), age (*P* < 0.01), years of education (*P* < 0.01), alcohol history (*P* < 0.05), hypertension (*P* < 0.01), and diabetes (*P* < 0.01) between POD and NPOD groups. The NPOD group has higher Aβ42 levels and lower T-tau and P-tau levels. In the POD group, there were more PA habits in the participants, and other special information was presented in Table 1.

**TABLE 1:** Demographic and characteristics of participants.

	NPOD	POD	*P*
Number	311	89	
Age, M (IQR), years	60 (52–65)	74 (70–78)	<0.001***
Sex (female/male)	182/129	61/28	0.088
Weight, M (IQR), kg	71 (62–78)	69 (60–77)	0.143
Education, M (IQR), years	11 (9–12)	7 (6–9)	<0.001***
Cigarette consumption, yes (%)	90 (28.9)	19 (21.3)	0.156
Alcohol habit, yes (%)	115 (37.0)	21 (23.6)	0.019*
Hypertension, yes (%)	108 (34.7)	50 (56.2)	<0.001***
Diabetes, yes (%)	46 (14.8)	26 (29.2)	0.002**
CHD, yes (%)	31 (10.0)	15 (16.9)	0.073
MMSE score, M (IQR)	28 (27–30)	29 (27–30)	0.312
**CSF biomarkers, M (IQR), pg/ml**			
Aβ42	350.28 (228.50–483.29)	308.84 (216.94–387.16)	0.004**
T-tau	117.99 (140.04–219.03)	199.92 (141.40–303.97)	0.006**
P-tau	38.38 (32.17–46.27)	42.20 (34.32–53.17)	0.004**
**Ratios of biomarkers, M (IQR)**			
Aβ42/T-tau	1.94 (1.35–2.82)	1.44 (0.87–2.26)	<0.001***
Aβ42/P-tau	8.85 (6.36–12.55)	7.31 (4.67–9.33)	<0.001***

Categorical variables are reported as numbers and percentages; continuous variables are reported as means ± SD, whereas non-normal data are expressed as the M (Q25, Q75). POD, postoperative delirium; NPOD, no postoperative delirium; MDAS, Memorial Delirium Assessment Scale; CHD, coronary heart disease; M, median; IQR, interquartile range. *P < 0.05; **P < 0.01; ***P < 0.001. The bold values are all *p* ≤ 0.05, statistically significant parameters.

### Protective and risk factors on POD

Binary logistics regression shows that PA [odds ratio (OR) = 0.589, 95% confidence interval (95 CI) 0.365–0.951, *P* < 0.05] (refer to Table 2 Model 1) is a protective factor for POD, as for CSF biomarker, Aβ42 is a protective factor for POD (OR = 0.992, 95 CI 0.987–0.996, *P* = 0.01), T-tau (OR = 1.009, 95 CI 1.005–1.014, *P* < 0.01), and P-tau (OR = 1.055, 95 CI 1.012–1.099, *P* < 0.05) are risk factors for POD (refer to Table 2 Model 2). The results show that it may be a significant protective factor if regular daily PA is carried out (OR = 0.336, 95 CI 0.158–0.717, *P* < 0.01) (refer to Table 3), and as we have mentioned above, Aβ42 is also a protective factor for POD (OR = 0.989, 95 CI 0.980–0.997, *P* < 0.01), T-tau (OR = 1.010, 95 CI 1.004–1.017, *P* < 0.01), and P-tau (OR = 1.061, 95 CI 1.003–1.123, *P* < 0.05) are risk factors for POD (refer to Table 3 Model 2).

**TABLE 2:** Logistic regression and sensitivity analysis in PNDABLE.

	Model 1		Model 2		Model 3		Model 4	
	OR (95% CI)	*P*	OR (95% CI)	*P*	OR (95% CI)	*P*	OR (95% CI)	*P*
Physical activity or not	0.336 (0.206–0.548)	**<0.001**	0.463 (0.271–0.791)	**0.005**	0.440 (0.244–0.793)	**0.006**	0.476 (0.255–0.887)	**0.019**
CSF biomarkers, median (IQR), pg/ml								
Aβ42	0.997 (0.996–0.999)	**0.001**	0.992 (0.987–0.996)	**0.001**	0.991 (0.986–0.996)	**0.001**	0.991 (0.985–0.997)	**0.002**
T-tau	1.006 (1.003–1.009)	**<0.001**	1.009 (1.005–1.014)	**<0.001**	1.011 (1.005–1.016)	**<0.001**	1.009 (1.004–1.015)	**<0.001**
P-tau	1.039 (1.018–1.059)	**<0.001**	1.055 (1.012–1.099)	**0.011**	1.057 (1.010–1.107)	**0.017**	1.060 (1.009–1.114)	**0.021**
Ratios of biomarkers, median (IQR)								
Aβ42/T-tau	0.601 (0.460–0.786)	**<0.001**	1.861 (1.193–2.902)	**0.006**	2.041 (1.243–3.350)	**0.005**	1.869 (1.132–3.086)	**0.014**
Aβ42/P-tau	0.862 (0.806–0.922)	**<0.001**	1.068 (0.883–1.292)	0.498	1.061 (0.860–1.309)	0.580	1.090 (0.870–1.365)	0.453

Model 1: the unadjusted logistic regression. Model 2: adjusted logistic regression. Model 3: first sensitivity analysis was based on more covariates including gender, years of education, and MMSE. Model 4: second sensitivity analysis was based on more covariates including gender, years of education, MMSE, history of alcohol consumption and smoking, diabetes, hypertension, and coronary heart disease. The bold values are all *p* ≤ 0.05, statistically significant parameters.

**TABLE 3:** Logistic regression analysis and sensitivity analysis for different frequencies of physical activity.

	Model 1		Model 2		Model 3		Model 4	
	OR (95% CI)	*P*	OR (95% CI)	*P*	OR (95% CI)	*P*	OR (95% CI)	*P*
A	0.275 (0.136–0.558)	**<0.001**	0.336 (0.158–0.714)	**0.005**	0.313 (0.141–0.696)	**0.004**	0.337 (0.143–0.795)	**0.013**
B	0.419 (0.191–0.919)	**0.030**	0.473 (0.203–1.103)	0.083	0.521 (0.206–1.315)	0.167	0.529 (0.197–1.419)	0.206
C	0.300 (0.086–1.053)	0.060	0.455 (0.116–1.782)	0.258	0.337 (0.077–1.481)	0.150	0.362 (0.079–1.657)	0.190
D	0.357 (0.169–0.753)	**0.007**	0.579 (0.256–1.310)	0.190	0.595 (0.244–1.451)	0.253	0.704 (0.279–1.775)	0.457

A: participate in physical activity daily. B: participate in physical activity multiple times a week. C: participate in physical activity once a week. D: occasionally participate in physical activity. Model 1: the unadjusted logistic regression. Model 2: adjusted more covariates logistic regression including Aβ42, T-tau, P-tau, Aβ42/T-tau, and Aβ42/P-tau. Model 3: first sensitivity analysis was based on more covariates including gender, years of education, and MMSE. Model 4: second sensitivity analysis was based on more covariates including gender, years of education, MMSE, history of alcohol consumption and smoking, diabetes, hypertension, and coronary heart disease. The bold values are all *p* ≤ 0.05, statistically significant parameters.

### Sensitivity analysis

In the end, there was only a slight change from the previous results, after two steps of sensitivity analysis, the CSF biomarkers were statically significant except Aβ42/P-tau (*P* = 0.453), but the conclusion that PA was a protective factor for POD remained (OR = 0.476, 95 CI 0.255–0.887, *P* < 0.05) (refer to Table 2 Model 4). We also made a sensitivity analysis of different PA frequencies and POD, as we expected, only the group that participated in PA daily still had prominent differences compared with others.

### Mediation analysis

**FIGURE 2:**
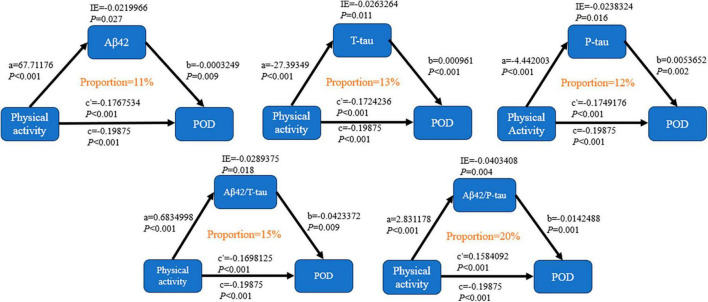
Mediation analyses with 10,000 bootstrapped iterations were used to examine the mediation effects of Aβ42, T-tau, P-tau, Aβ42/T-tau, and Aβ42/P-tau on POD.

Mediator effect analysis showed that PA might reduce the incidence of POD through CSF biomarkers, such as Aβ42 (proportion: 11%, *P* < 0.05), T-tau (proportion: 13%, *P* < 0.05), and P-tau (proportion: 12%, *P* < 0.05). The relevant demerits are displayed in Figure 2.

### *Post hoc* analyses

After excluding a part of patients with MMSE > 28, diabetes, and coronary heart disease, a replication of the PNDABLE cohort study was performed. The results are as follows: Model 1 [PA (OR: 0.366, 95 CI 0.185–0.722, *P* < 0.01)]; Model 2 [PA (OR: 0.534, 95 CI 0.290–0.984, *P* < 0.05)]; Model 3 [PA (OR: 0.474, 95 CI 0.265–0.849, *P* < 0.05)], it shows that our results are still stable, which means PA is a protective factor for POD (Table 4).

**TABLE 4:** *Post hoc* analyses in the PNDABLE study.

	Model 1		Model 2		Model 3	
	OR (95% CI)	*P*	OR (95% CI)	*P*	OR (95% CI)	*P*
PA	0.366 (0.185–0.722	**0.004**	0.534 (0.290–0.984)	**0.044**	0.474 (0.265–0.849)	**0.012**
Aβ42, pg/ml	0.989 (0.982–0.995)	**<0.001**	0.991 (0.986–0.997)	**0.002**	0.991 (0.986–0.996)	**0.001**
T-tau, pg/ml	1.010 (1.004–1.016)	**0.002**	1.009 (1.003–1.014)	**0.002**	1.011 (1.006–1.016)	**<0.001**
P-tau, pg/ml	1.096 (1.037–1.157)	**0.001**	1.056 (1.008–1.107)	**0.022**	1.053 (1.009–1.099)	**0.018**
Aβ42/T-tau	2.163 (1.253–3.733)	**0.006**	1.951 (1.210–3.144)	**0.006**	1.841 (1.131–2.994)	**0.014**
Aβ42/P-tau	1.163 (0.916–1.476)	0.215	1.050 (0.849–1.298)	0.652	1.086 (0.885–1.332)	0.431

OR, odds ratio; 95% CI, 95% confidence interval. a Model 1: exclude MMSE <28. b Model 2: exclude diabetes. c Model 3: exclude coronary heart disease. The bold values are all *p* ≤ 0.05, statistically significant parameters. Model 1–3 refers to the model of Hoc Analyses.

## Discussion

The results of this study suggest that PA is a protective factor for POD and may play a mediating role through CSF biomarkers (Aβ42, T-tau, and P-tau).

Postoperative delirium is an acute brain function impairment with adverse consequences such as decreased organ function and impairment of attention, memory, and spatial abilities. Previous studies have found that POD is closely related to CSF biomarkers (Körtvélyessy et al., 2015), Aβ is a product of amyloid precursor proteolysis, its plaques can cause oxidative stress and mitochondrial damage (Lin et al., 2023); tau protein is a microtubule-associated protein in the brain that is essential for the formation and stabilization of micro crowns which can aggregate to form pathological tangles (Selkoe and Hardy, 2016; Horie et al., 2021), and the plaques formed by Aβ bind to phosphorylated tau in cells, leading to the death of neurons (Polanco et al., 2018). Our study showed that the elevated CSF Aβ42, Aβ42/T-tau, and Aβ42/P-tau may be protective factors for POD in the patients, which can predict the occurrence of POD, and Aβ42 may be the major component of senile plaques in some Neurological disorders, as we mentioned POD in this study and the study of AD in Selkoe and Hardy (2016).

Obviously, a large number of studies have been reported on biomarkers and POD at present, but there is no effective measure to intervene in the increase or decrease of CSF biomarkers to improve the occurrence of POD. However, previous research in our group has found that the occurrence of POD may be reduced after adjusting lifestyle habits such as tea drinking and drinking (Lin et al., 2022; Wu et al., 2023b). As a well-known healthy lifestyle habit, it is reasonable to speculate that PA may be related to the occurrence of POD. In recent years, the progress and adhibition of PA therapy in clinical and recovery have been conspicuous, PA has a positive effect on many diseases (Fukushima et al., 2023; Sanchez-Lastra et al., 2023; Zare et al., 2023), dementia is also included (Verghese et al., 2003), others include managing chronic pain and improving the prognosis of patients with neurological diseases (Tollár et al., 2020; Loftus et al., 2022; Sanchez-Lastra et al., 2023). The results of this study found out that PA may be another protective factor for POD in the patients, and the results remained robust after adjusting for confounders. The reason why PA is beneficial to POD may be that cardiopulmonary function is related to local brain volume, gray matter and white matter integrity, and the increase in gray matter and white matter volume in the prefrontal and temporal cortex of the patients after PA may be a mechanism for PA to affect the occurrence of POD (Colcombe et al., 2006; Hortobágyi et al., 2022). In addition, PA can induce the construction of neurons, and at the same time, the brain is able to take up lactic acid produced by muscle during PA, which is used to increase neuronal excitability and β amyloid clearance (Hillman et al., 2008; Hortobágyi et al., 2022). It has been reported that PA improves cognitive function by enhancing the ability of individuals to respond to new demands through individual behavior, and the mechanism of beneficial cognitive function may also be related to synaptogenesis, angiogenesis, and neurotrophic factor release (Hillman et al., 2008; Hötting and Röder, 2013).

In previous studies, PA has been linked to decreasing the incidence of cognitive decline and dementia (Verghese et al., 2003). The research in this paper has increased the role of PA in reducing the incidence of POD. In order to further investigate the relationship between PA and POD, we added some confounding factors, the results show that Aβ42 plays a protective role in the occurrence of POD, while T-tau and P-tau play dangerous roles, and it corroborates the results of our group’s previous research (Wu et al., 2023b). The results were further validated in the groups of patients with MMSE > 28, diabetes, and coronary heart disease. Furthermore, we grouped participants’ PA frequencies concerning Zhong et al.’s (2022) study. The results were similar to what we had predicted, only by consistently engaging PA daily could it be considered a significant protective factor for POD, which is similar to the conclusions reached by the previous study (Zhong et al., 2022). The effect of frequency on the incidence of POD is apparent (Lee et al., 2019; Zhong et al., 2022), so does the time of day when PA occurs also have an impact on the occurrence of POD? It is a pity that we only find out previous studies have shown that PA earlier in the morning is beneficial in reducing the risk of hypertension for the different timing of PA (Li et al., 2023), but it has not been shown whether different PA times of day have a significant difference in the occurrence of POD, and we speculate that different PA times of day and PA intensity may also affect POD, so further research is needed to confirm this hypothesis.

Even though the relationship between POD and PA seems to be obvious, how to mediate POD for PA needs to be further studied. Based on the results above, we have applied a hypothesis that POD might mediated by PA via CSF biomarkers. Therefore, our study of mediation effect analysis conducted a preliminary exploration of the possible potential mechanisms of POD. The results showed that the development of POD could be reduced possibly through the CSF biomarkers.

The novelty of this article is that it has explored the relationship between PA and frequency and the occurrence of POD, possibly through CSF biomarkers. We speculate that there may also be a link between PA and POD, and Alzheimer’s and postoperative delirium may share some of the same mechanism (Querfurth and LaFerla, 2010; Selkoe and Hardy, 2016). At the same time, a growing body of research supports PA as a lifestyle factor that may lead to lifelong increases in physical and mental health (Fukushima et al., 2023). In addition, in order to further explore the mechanism of how PA mediates the occurrence of POD, we performed a mediating effect analysis, and the results showed that the incidence of POD may be mediated in part through biomarkers.

However, there is no doubt that there are certain limitations to our research. First of all, the number of cases we studied is small, and further in-depth clinical studies are needed. Second, only the frequency of PA is counted, the intensity and the chronoactivity pattern of activity may also be an important factor. Third, this is a single-center study, and a multicenter study is still necessary. Finally, only a portion of CSF biomarkers have been studied in the mechanism of PA and POD, and the next step should be to continue to explore whether PA plays a role through other bioindicators.

## Conclusion

In conclusion, PA may be a protective factor for POD and might work through Aβ42, T-tau, and P-tau. Patients who participate in PA have a lower incidence of POD. This study provides a new perspective on the prevention of POD and lessens the occurrence of POD through lifestyle changes. Taking precautions against POD may be guided by developing PA habits.

## Data availability statement

The original contributions presented in this study are included in this article/supplementary material, further inquiries can be directed to the corresponding author.

## Ethics statement

The studies involving humans were approved by the Ethics Committee of Qingdao Municipal Hospital. The studies were conducted in accordance with the local legislation and institutional requirements. The participants provided their written informed consent to participate in this study.

## Author contributions

JK: Writing – original draft, Writing – review & editing. XL: Writing – review & editing. BW: Writing – review & editing. SX: Writing – original draft. YW: Writing – original draft. SH: Writing – original draft. HG: Writing – review & editing. RD: Writing – review & editing. YL: Writing – review & editing. CL: Writing – original draft. YB: Writing – review & editing.
